# The Effects of Persistent Olfactory and Gustatory Dysfunctions on Quality of Life in Long-COVID-19 Patients

**DOI:** 10.3390/life12020141

**Published:** 2022-01-19

**Authors:** Luigi Angelo Vaira, Claudia Gessa, Giovanna Deiana, Giovanni Salzano, Fabio Maglitto, Jerome R. Lechien, Sven Saussez, Pasquale Piombino, Andrea Biglio, Federico Biglioli, Paolo Boscolo-Rizzo, Claire Hopkins, Valentina Parma, Giacomo De Riu

**Affiliations:** 1 Maxillofacial Surgery Operative Unit, Department of Medical, Surgical and Experimental Sciences, University of Sassari, 07100 Sassari, Italy; c.gessa@studenti.uniss.it (C.G.); andreabiglio@gmail.com (A.B.); gderiu@uniss.it (G.D.R.); 2 Biomedical Science Department, PhD School of Biomedical Science, University of Sassari, 07100 Sassari, Italy; 3 Direction, Hygiene and Hospital Infection Control Operative Unit, Department of Medical, Surgical and Experimental Sciences, University of Sassari, 07100 Sassari, Italy; giovanna.deiana90@gmail.com; 4 Maxillofacial Surgery Department, University Hospital of Naples “Federico II”, 80131 Naples, Italy; giovannisalzanomd@gmail.com (G.S.); fmaglitto@gmail.com (F.M.); piombino@unina.it (P.P.); 5 Department of Human and Experimental Oncology, Faculty of Medicine UMONS Research Institute for Health Sciences and Technology, University of Mons (UMons), 7000 Mons, Belgium; jerome.lechien@umons.ac.be (J.R.L.); sven.saussez@umons.ac.be (S.S.); 6 Department of Otolaryngology-Head & Neck Surgery, Foch Hospital, School of Medicine, UFR Simone Veil, Versailles Saint-Quentin-en-Yvelines University, Paris Saclay University, 91190 Paris, France; 7 Maxillofacial Surgery Department, San Paolo Hospital, ASST Santi Paolo e Carlo, University of Milan, 20122 Milan, Italy; federico.biglioli@unimi.it; 8 Department of Medicine, Surgery and Health Sciences, University of Trieste, 34149 Trieste, Italy; pboscolorizzo@yahoo.it; 9 Guy’s and St Thomas NHS Foundation Trust, London SE1 7EH, UK; clairehopkins@yahoo.com; 10 British Rhinological Society (President), London WC2A 3PE, UK; 11 Monell Chemical Senses Center, Philadelphia, PA 19104, USA; vparma@monell.org; 12 Department of Psychology, Temple University, Philadelphia, PA 19122, USA

**Keywords:** smell, taste, olfactory disorders, gustatory disorders, anosmia, ageusia, long-COVID-19, quality of life, SARS-CoV-2

## Abstract

(1) Background: Persistent olfactory (POD) and gustatory (PGD) dysfunctions are one of the most frequent symptoms of long-Coronavirus Disease 2019 but their effect on the quality of life (QoL) of patients is still largely unexplored. (2) Methods: An online survey was administered to individuals who reported to have had SARS-CoV-2 infection at least 6 months prior with persisting COVID-19 symptoms (using the COVID symptom index), including ratings of POD and PGD, and their physical (PCS) and mental (MCS) components of quality of life were assessed using the standardized short form 12 questionnaire (SF-12). (3) Results: Responses from 431 unique individuals were included in the analyses. The most frequent persistent symptoms were: fatigue (185 cases, 42.9%), olfactory dysfunction (127 cases, 29.5%), gustatory dysfunction (96 cases, 22.3%) and muscle pain (83 cases, 19.3%). Respondents who reported persisting muscle pain, joint pain, fatigue, headache, gastrointestinal disturbances, and dyspnea had significantly worse PCS. Those experiencing persistent fatigue and dyspnea also showed significantly lower MCS. Respondents reporting POD or PGD showed significantly worse QoL, but only pertaining to the MCS. Multiple regressions predicted MCS based on olfactory and marginally on gustatory ratings, but not PCS. Age significantly affected the prediction of PCS but not MCS, and gender and temporal distance from the COVID-19 diagnosis had no effect. (4) Conclusions: POD and PGD are frequent symptoms of the long-COVID-19 syndrome and significantly reduce QoL, specifically in the mental health component. This evidence should stimulate the establishment of appropriate infrastructure to support individuals with persistent CD, while research on effective therapies scales up.

## 1. Introduction

Persistent olfactory (POD) and gustatory (PGD) dysfunctions, together known as chemosensory dysfunction (CD), are among the most frequent symptoms of long-Coronavirus Disease 2019 (long-COVID-19) [1]. The first studies with follow-up at 6 [2,3,4,5,6,7,8,9] and 12 months [10,11,12] show high prevalence of persistent CD in up to 67% of participants who had a symptomatic infection with severe acute respiratory syndrome coronavirus 2 (SARS-CoV-2). Given the high prevalence of CD in individuals with first-time COVID-19, their high frequency in individuals experiencing reinfections [13,14] and in those vaccinated against COVID-19 [15], CD will represent a serious health problem in the near future. Unlike other sensory deficits, the impact of CD on the individual’s well-being is often overlooked or minimized by those who do not routinely deal with these pathologies [16]. Nonetheless, the integrity of the olfactory and gustatory perception provides key support to well-being significantly impacting nutrition, social behavior and the ability to protect oneself from environmental dangers [17].

Despite the presence of CD having been deemed a favorable prognostic factor for the acute phase of COVID-19 [18,19,20], CD is associated with increased levels of anxiety and depression [21,22]. While there is sufficient evidence to indicate that CD is associated with reduced quality of life (QoL) [23,24,25], on the other hand the effects of CD on the QoL of patients with long-COVID-19 remain significantly underexplored. Burges Watson et al. [26] conducted a text analysis of posts generated by 9000 users on a Facebook group that brought together individuals with COVID-19-related CD. Users with CD reported a broad spectrum of circumstances that reduced their wellbeing, such as work or study difficulties, impaired eating with loss of appetite and weight changes, social and interpersonal limitations. Additionally, Ohla and colleagues [27] in their pre-print suggest that POD in the acute phase of the disease is associated with more COVID-19 symptoms overall and may represent a key marker of long-COVID-19.

A recent review of the literature [28] looking for studies on the impact of CD on QoL in COVID-19 found only four studies published so far [29,30,31,32]. These studies share some limitations which call into question the robustness of the conclusions made. First, the use of validated QoL questionnaires is essential to obtain reliable data and provide meaningful comparisons with normative groups [33], but three out of those four studies based their conclusions only on self-reported QoL [29,30,31]. Second, all studies investigate QoL at less than 3 months from COVID-19 diagnosis, a time period in which there are high rates of spontaneous recovery and the full impact of CD on well-being may not be apparent. More appropriate follow-up time points are a minimum of 6 months [7] to more than a year [34]. As a result, our knowledge on the relationship between QoL and CD in long-COVID-19 is limited. For instance, the relationship between QoL and long-COVID-19 has so far been characterized from the perspective of patient characteristics, rather than the persistence of symptoms [35]. In this case, a meta-regression analysis of seven studies shows that poor QoL measured via self-reports was significantly higher among individuals post-COVID-19 who reported persistent fatigue, but not CD [35].

To fill this gap here we investigate the associations between physical- and mental-health-related QoL scores (obtained via the standardized measure SF-12 [36,37]) and the persistence of various symptoms at least 6 months after the diagnosis of SARS-CoV-2 infection. We hypothesize that individuals with persistent symptoms will report poorer physical and mental QoL at more than 6 months from COVID-19 diagnosis as compared to individuals who resolved all symptoms within that timeframe. We expect POD and PGD to have a similar adverse impact on mental health-related QoL, although not on physical health-related QoL. We expect such an impact on mental QoL to be significantly greater than in the general population. We anticipate respondents who endure persistent symptoms for longer to report worse mental QoL.

## 2. Materials and Methods

An online survey [38] in Italian was disseminated throughout Italy from May to August 2021 through social networks. Individuals who reported having had SARS-CoV-2 infection, confirmed by nasopharyngeal swab, at least 6 months prior were included in the study. Individuals with pre-existing history of CD or other pathologies known to affect CD or QoL (i.e., head trauma, surgery or radiotherapy of the craniofacial region, psychiatric or neurological disease) were excluded from the analysis.

In the first part of the survey, demographic information (only age and sex) and the inclusion criteria were assessed. On the basis of the COVID-19 symptom index [39], information was collected on which symptoms were present during the active COVID-19 infection and if any symptom was still present at the time of completion of the questionnaire, at least 6 months following initial COVID-19 diagnosis. All COVID-19 symptoms were assessed as binary responses (i.e., present or absent). Respondents were also asked to rate olfactory and gustatory function, both during COVID-19 and at the time of completion of the survey, with a visual analogue scale (VAS) ranging from 0 (perception completely absent) to 10 (typical perception). Finally, the Italian version of the SF-12 questionnaire [40] was administered to the respondents.

SF-12 [36,37] is a validated and widely used instrument that provides a self-reported outcome measure of individuals’ QoL through eight domains: (i) limitations in physical activities due to health problems; (ii) limitations in social activities due to physical or emotional problems; (iii) limitations in usual role activities because of physical health problems; (iv) bodily pain; (v) general mental health; (vi) limitations in usual role activities due to emotional problems; (vii) vitality; and (viii) general health perceptions. The questionnaire allows to obtain two scores ranging from 0 to 100: the physical component summary (PCS) and the mental component summary (MCS), which represent an index of individual physical and mental QoL, respectively. The PCS and the MCS scores are transformed to have a mean of 50 and a standard deviation of 10. Thanks to this standardization, scores greater than or equal to 50 are directly interpreted as QoL above that reported by the average of the general population and scores below 50 are directly interpreted as QoL below that reported by the average of the general population [36,37,41].

The study was conducted in accordance with the Declaration of Helsinki and adhered to Good Clinical Practice guidelines. Approval for this exempt protocol for anonymized data collection was obtained from the University Hospital of Cagliari Ethics committee (approval no. 2021/7118—28 April 2021).

### Statistical Analysis

Statistical analyses were performed using R 3.6.3 and RStudio 1.3.952 (Boston, MA, USA). Categorical variables are reported as frequencies and/or percentages. Descriptive statistics for quantitative variables are reported as the mean ± standard deviation (SD) or median (interquartile range [IQR]). The Mann–Whitney U test was performed to evaluate the statistical significance of differences in PCS and MCS between two groups of respondents: those whose symptoms persist more than 6 months after the initial COVID-19 diagnosis, and those whose symptoms have remitted within that timeframe. One sample *t*-tests with mu = 50 were run to determine whether PCS and MCS in respondents reporting POD or PGD were significantly different from the general population. To reduce the magnitude of type II statistical error, the analysis was conducted only for symptoms that persisted in at least 32 respondents. This sample size was identified by using G*power 3.1 (Heinrich Heine University Dusseldorf, Dusseldorf, Germany) and considering 0.5 Cohen’s D, 80% power and 10% margin of error. We ran separate multiple regression models via the *lme4* package to predict PCS and MCS based on olfactory and gustatory VAS ratings, age sex, and time from COVID-19 diagnosis. For all analyses, the level of statistical significance was set at *p* < 0.05 with a 95% confidence interval.

## 3. Results

From 18 May to 18 August 2021, we collected 470 complete responses (63 incomplete responses were excluded) to the survey. Of these, 39 responses were not considered for analysis as they did not meet the inclusion criteria: absence of a confirmed diagnosis of infection (*n* = 23), less than 6 months follow-up (*n* = 10), previous CD or severe comorbidities (*n* = 6). The final analyses were run on 431 responses from unique individuals.

The sample included 329 females (76.3%) and 102 males (23.7%) with a mean age of 38.4 ± 12.5 years old (range 12–71 years). The mean distance from diagnosis of SARS-CoV-2 infection was 253.4 ± 70.5 days (range 181–486 days). At the time of completion of the survey (at least 6 months after infection), 73.3% of respondents had at least one symptom persisting from the initial COVID-19 diagnosis (Table 1).

The most frequent persistent symptoms were: fatigue (185 cases, 42.9%), olfactory dysfunction (127 cases, 29.5%), gustatory dysfunction (96 cases, 22.3%) and muscle pain (83 cases, 19.3%). Specifically, of the 306 patients who self-reported olfactory dysfunction during infection, 41.4% reported POD at the time of completing the questionnaire. The frequency of PGD among patients who self-reported olfactory dysfunction during infection was instead of 34.8%. Table 2 details the frequency of chemosensory symptoms reported by respondents.

The POD reported were anosmia in 18 (4.2%) and hyposmia in 109 cases (25.2%). Among respondents with a persistent olfactory dysfunction, 87.4% reported an associated qualitative dysfunction (e.g., parosmia or phantosmia). As for PGD, 12 respondents (2.8%) reported ageusia, while hypogeusia was detected in 84 cases (19.5%). Among respondents with PGD, 93.7% reported dysgeusia in association with qualitative taste dysfunction.

The analysis of the effects of the persistence of one of the symptoms on QoL indices was carried out for all symptoms reported by at least 32 respondents as still present >6 months post-acute COVID-19 infection. A summary of the results of the analysis is shown in Table 3. Respondents reporting at least one persistent symptom had significantly worse PCS (symptomatic vs. asymptomatic: 59 (53.9–61.3) vs. 51.5 (42.3–56.9), *p* < 0.001) and MCS (symptomatic vs. asymptomatic: 50.1 (45.8–54.9) vs. 45.9 (36.7–52.1), *p* < 0.001) than respondents who resolved all symptoms at the time of completion of the survey.

Specifically, respondents who reported persisting muscle pain, joint pain, fatigue, headache, gastrointestinal disturbances, and dyspnea had significantly worse PCS than respondents for whom these symptoms had completely regressed (Table 3). Those experiencing persistent fatigue and dyspnea also showed significantly lower MCS than respondents who reported to have completely healed (Table 3).

**Figure 1:**
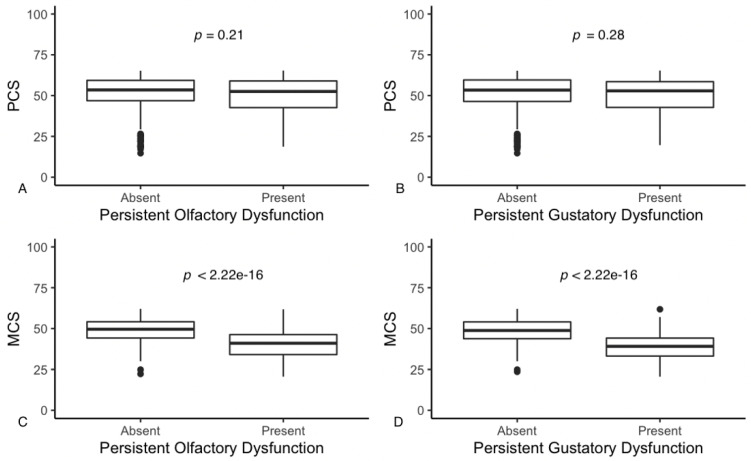
Results of the analysis of the effects of POD (**A**,**C**) and PGD (**B**,**D**) on the QoL of respondents by mental and physical component scores. The shaded rectangle identifies the IQR around the median, which corresponds to the horizontal bold line within the rectangle. The error bars identify the maximum and minimum values. Respondents reporting CD for at least 6 months post-COVID-19 diagnosis showed significantly worse mental QoL than respondents who reported that CD had completely regressed.

Respondents reporting CD for at least 6 months post-COVID-19 diagnosis showed significantly worse mental QoL (but not physical) than respondents who reported that CD had completely regressed (Table 3 and Figure 1). When comparing the PCS of respondents with POD and PGD with the general population (mean = 50 and sd = 10), respectively, no significant differences emerge (POD: *t* = −0.78, df = 126, *p* = 0.44; PGD: *t* = −0.67, df = 95, *p* = 0.51) (Figure 1A,B). However, MCS in both respondents with POD and PGD is worse than in the general population (POD: *t* = −12.2, df = 126, *p* < 2.2 × 10^−16^; PGD: *t* = −12.6, df = 95, *p* < 2.2 × 10^−16^) (Figure 1C,D).

**Figure 2:**
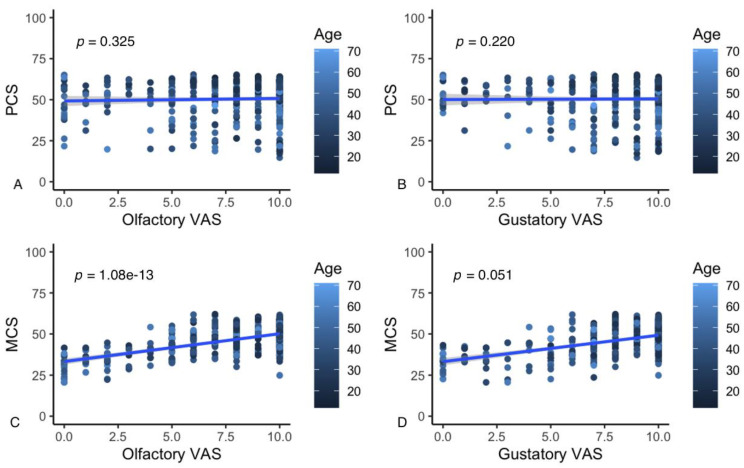
Results of the correlation analysis between olfactory and gustatory VAS scores and PCS (**A**,**B**) and MCS (**C**,**D**).

Multiple regression analyses indicate that the olfactory VAS rating (*t* = 7.63, df = 425, *p* = 1.5 × 10^−13^) and nominally the gustatory VAS rating (*t* = 1.95, df = 425, *p* = 0.05) predict the MCS score. Age, sex and temporal distance from the COVID-19 acute diagnosis were not significant factors in this prediction (Figure 2). Only age had a significant effect in the prediction of PCS (*t* = −7.45, df = 425, *p* = 5.32 × 10^−13^), in that younger participants report better PCS.

## 4. Discussion

The SARS-CoV-2 pandemic helped put taste and smell disorders in the headlines [42,43,44,45,46]. These pathologies were the prerogative of a narrow circle of specialists and mostly unknown to the lay public. Even before the pandemic, viral infections were the most frequent cause of persistent anosmia [47], yet the COVID-19 pandemic significantly increases the magnitude of this phenomenon. First, due to the very high number of patients with POD and PGD: up to 67% of all those who have had symptomatic COVID-19 [2,3,4,5,6,7,8,9,10,11,12,48,49,50,51,52,53]. Moreover, CD are also proving a frequent symptom in reinfections [13,14] and in COVID-19 in vaccinated individuals [15]. It has not yet been clarified what the frequency of persistent disturbances is in these groups of individuals, but if the prevalence recorded in cases of primary infection were confirmed, it would mean that this problem may not end with the immunization of the population. Second, the identification of the pathogenesis [54,55,56,57,58,59,60,61] and risk factors [2,3,4,5,6,7,8,9,10,11,12,62,63,64,65,66,67,68] for the development of persistent CD are in its infancy. For this reason, no effective therapies have yet been found for the prevention and treatment of COVID-19 related POD and PGD [69,70,71,72,73,74,75].

In the present study, POD and PGD were confirmed as one of the most frequent symptoms of long-COVID-19 with a prevalence of 24.6% and 19.5%, respectively, second only to fatigue (42.5%), a much more aspecific symptom [76]. In almost all cases, quantitative CD (i.e., anosmia/hyposmia, and ageusia/hypogeusia) was associated with qualitative CD (i.e., parosmia or dysgeusia) >6 months after diagnosis. Qualitative olfactory dysfunctions generally arise 2–3 months after CD onset [77] and are due to aberrant regeneration of the connections between olfactory neurons and higher brain areas [78]. Overall, qualitative disorders have been more associated with severe reduction in QoL than purely quantitative disorders [79].

The presence of at least one persistent symptom was shown to be related to significantly lower PCS and MCS values compared to respondents who resolved all symptoms >6 months after COVID-19 diagnosis. In particular, all symptoms that affect the respondent’s ability to carry out physical activities normally (i.e., fatigue, dyspnea, muscle and joint pain) were associated with a significant reduction in PCS. POD and PGD were instead accompanied by a significant reduction in MCS alone. Such a reduction in the mental component of QoL is greater than that reported for pathologies, such as chronic renal failure requiring long-term dialysis [80], chronic ischemic heart disease [81] and oncological problems [82]. The strength of the relationship between POD or PGD and MCS is underlined by the strong and significant relationship detected between self-reported VAS assessment of smell and, marginally, of taste and MCS values, the net effects of age, sex and the duration of the disease (Figure 2).

Older respondents in our sample reported lower PCS. Interestingly, physically impacting symptoms, such as muscle and joint pain, were not accompanied by significant reductions in MCS. This could be linked to the fact that over time the symptoms that cause physical limitations tend to have a less marked impact on the psychological component of the individual who can, therefore, return to acceptable MCS values [83].

Such an adaptation effect is not true for persistent CD, which is associated with severe psychological consequences even long after disease onset. Indeed, 100 days post-COVID-19 self-identification, the risk for individuals with CD to transition to depression or a suicidal ideation risk state is greater than for individuals without CD [22]. This finding underlines the importance of taste and smell as a determinant of the mental QoL of the individual as their integrity is crucial for the thriving of vital functions [17,74,84]. Importantly, the duration of the pandemic provides a natural constraint within which to assess the effects of CD on QoL. Nonetheless, adverse consequences on nutritional and mental health are expected to snowball over the years, and the healthcare system needs to build the infrastructure to address these issues, including support services as well as the development of therapies to cure CD for the millions of individuals battling long-COVID. The lack of monitoring on emerging CD at the beginning of the pandemic has already represented a lost opportunity to implement effective public health decisions [85].

This study has some limitations. First, it was not possible to perform a psychophysical evaluation of smell and taste; the self-reported evaluation of olfactory and gustatory loss and recovery alone can introduce bias both in terms of the underestimation of the loss and overestimation of recovery [86,87,88]. Second, respondents were asked to report symptoms that occurred during the infection that was active at least 6 months prior to engaging with the survey, and this may have introduced recall biases [89]. Third, in order to avoid an overestimation of persistent symptoms and to be able to count on reliable prevalence, the survey was not disseminated through direct channels with groups of individuals affected by long-COVID-19, but was addressed to anyone who had been infected with SARS-CoV-2 in the previous 6 months. However, it is possible that respondents with persistent symptoms were more motivated to respond to the survey. Additionally, given the overlap between respondents reporting simultaneously quantitative and qualitative POD and PGD, it was not possible to address the effect of these disorders separately on QoL. Finally, voluntary recruitment may have introduced selection biases [89].

## 5. Conclusions

POD and PGD are a frequent symptom of the long-COVID-19 syndrome and significantly reduce QoL, specifically in the mental health component. This evidence should stimulate the establishment of appropriate infrastructure to support individuals with persistent CD, while research on effective therapies scales up. In particular, these patients should be managed by multidisciplinary teams that include psychiatrists and nutritionists as well as specialists in the treatment of CD. The results of this study help to underline the impact that POD and PGD are having on these individuals and should push us to focus research on the risk factors that identify COVID-19 patients at risk of developing a persistent disorder. The persistence of a long-term CD can in fact be a source of depression, eating disorders and social isolation [17,21,22,23,24,25]. Therefore, it is important to treat these patients early to avoid these important psychological morbidities and because the regression of the disorder is all the more difficult the longer the treatment is delayed [71,73,74].

## Figures and Tables

**Table 1:** Survey-based frequency of Coronavirus Disease 2019 (COVID-19) symptoms during acute infection and at >6 months post-acute COVID-19 infection.

Symptom	During Acute COVID-19 Infection *n* (%)	>6 Months Post-Acute COVID-19 Infection *n* (%)
Fever	280 (65%)	3 (0.7%)
Muscle pain	287 (66.6%)	83 (19.3%)
Joint pain	244 (56.6%)	76 (17.6%)
Cough	215 (49.9%)	16 (3.7%)
Fatigue	325 (75.4%)	185 (42.9%)
Headache	285 (66.1%)	65 (15.1%)
Gastrointestinal symptoms	169 (39.2%)	32 (7.4%)
Olfactory dysfunction	306 (71%)	127 (29.5%)
Gustatory dysfunction	276 (64%)	96 (22.3%)
Dyspnea	120 (27.8%)	46 (10.7%)
Nasal obstruction	155 (36%)	19 (4.4%)
Conjunctivitis	44 (10.2%)	12 (2.5%)
No symptom	10 (2.3%)	110 (25.5%)

**Table 2:** Survey-based frequency of specific olfactory and gustatory dysfunctions during acute infection and at >6 months post-acute COVID-19 infection.

	During Acute SARS-CoV-2 Infection *n* (%)	>6 Months Post-Acute SARS-CoV-2 Infection *n* (%)
**Olfactory dysfunction**		
Anosmia	238 (55.2%)	18 (4.2%)
Hyposmia	68 (15.8%)	109 (25.2%)
Normal	125 (29%)	304 (70.5%)
Parosmia	41 (9.5%)	96 (22.3%)
Phantosmia	15 (3.5%)	28 (6.5%)
VAS self-assessment (0–10, mean ± SD)	3.6 ± 4.2	7.8 ± 3
**Gustatory dysfunction**		
Ageusia	198 (46%)	12 (2.8%)
Hypogeusia	78 (18.1%)	84 (19.5%)
Normal	155 (36%)	335 (77.7%)
Dysgeusia	53 (12.3%)	90 (20.9%)
VAS self-assessment (0–10, mean ± SD)	4.3 ± 4.2	8.3 ± 2.8

**Table 3:** Physical (PCS) and mental quality of life (QoL) (MCS) scores by presence of COVID-19 symptom persisting for >6 months post-acute COVID-19 diagnosis.

Symptom Persisting at >6 Months Post-Acute COVID-19 Diagnosis	Absent (Median (IQR))	Present (Median (IQR))	Mann–Whitney *p*-Value
**Muscle pain**			
PCS	55.1 (49.7–60.3)	37.8 (25.2–48.5)	<0.001
MCS	47.1 (41.3–53.2)	47.1 (40.9–52.6)	0.331
**Joint pain**			
PCS	55.3 (49.6–60.4)	37.6 (25.2–47.4)	<0.001
MCS	47.2 (41.3–52.9)	47.1 (41–53.6)	0.762
**Fatigue**			
PCS	57.1 (52.5–61.1)	46.3 (34.9–53.2)	<0.001
MCS	47.6 (41.8–53.3)	46.3 (39.8–52.5)	0.048
**Headache**			
PCS	54.6 (47.6–60.1)	46.1 (34.7–52.6)	<0.001
MCS	47.2 (41.5–53)	46 (39.3–53.9)	0.554
**Gastrointestinal disorders**			
PCS	53.7 (46.5–59.8)	46.5 (28.2–53.2)	<0.001
MCS	47.2 (41.4–53.1)	45.7 (40.8–52.8)	0.429
**Olfactory dysfunction**			
PCS	53.4 (46.5–59.4)	52.5 (42.4–59.1)	0.207
MCS	49.6 (44.2–54.2)	41 (33.9–46.3)	<0.001
**Gustatory dysfunction**			
PCS	53.4 (46.4–59.6)	52.9 (42.5–58.5)	0.282
MCS	48.8 (43.7–54.1)	39.1 (33–44.2)	<0.001
**Dyspnea**			
PCS	53.9 (47.6–59.9)	39.9 (29.3–51.8)	<0.001
MCS	47.8 (42.1–53.4)	38.2 (33.6–44.6)	<0.001

## Data Availability

L.A. Vaira and C. Gessa had full access to all data in the study and take responsibility for the integrity of the data and the accuracy of the data analysis.
